# Evaluation of advanced life support training through virtual reality: A cross-sectional observational study in a hypothermia and helicopter evacuation scenario

**DOI:** 10.1097/MD.0000000000047135

**Published:** 2026-01-16

**Authors:** Marina Sánchez Gómez, Carmen Amalia López López, Robert Greif, Federico Semeraro, Ana Belén Ocampo Cervantes, Manuel Pons Claramonte, Sergio Nieto Caballero, Petronila Mireia Alcazar Artero, Manuel Pardo Rios, Daniel Guillén Martínez

**Affiliations:** a Grupo de Investigación de Nuevas Tecnologías para la Salud, Nursing Factulty, UCAM Universidad Católica de Murcia, Región de Murcia, Spain; b Gerencia de Urgencias y Emergencias Sanitarias 061 de la Región de Murcia, Murcia, Spain; c Faculty of Medicine, University of Bern, Bern, Switzerland; d Department of Surgical Science, University of Torino, Torino, Italy; e Department of Anaesthesia, Intensive Care and EMS, Maggiore Hospital Bologna, Bologna, Italy.

**Keywords:** advanced life support, clinical simulation, educational assessment, hypothermia, medical education, virtual reality

## Abstract

To evaluate clinical performance and user perception after advanced life support training through immersive virtual reality (VR) simulation, focused on decision-making in a hypothermia scenario involving a helicopter evacuation. Cross-sectional observational study with an analytical component. Update Course on Ultrasound and Emergencies organized by the semFYC, held in Las Palmas de Gran Canaria from February 6 to 8, 2025. 101 healthcare professionals who fully completed the simulation experience and assessment questionnaires. Immersive VR clinical simulation using a 360° video recorded at a ski station, presented through Meta Quest 3® headsets. The scenario included 16 clinical decisions related to the management of a hypothermic patient during air transport. Percentage of correct clinical decisions, subjective perception (satisfaction, realism, confidence), and system usability assessed using the System Usability Scale (SUS). The mean score for correct decisions was 69.4% (SD = 15.2). Participants rated the experience very positively, highlighting realism (8.87/10), training usefulness (M > 4.7/5), and increased clinical confidence. The mean SUS score was 76.7, indicating “good” usability. A lower performance was identified in specific advanced hypothermia decision-making items. Immersive VR simulation proved to be a feasible, well-accepted, and useful strategy for training in clinical decision-making in complex, low-frequency scenarios. The results support its integration as a complementary tool in continuing emergency training programs.

## 1. Introduction

Cardiopulmonary resuscitation (CPR) is an essential skill in emergency medical care, and when applied early and with quality, significantly improves survival after cardiorespiratory arrest.^[1,2]^ The European Resuscitation Council (ERC) guidelines underline the need for continuous training that is structured and evidence-based so that all health professionals maintain these skills at optimum levels over time.^[3]^

Traditionally, the teaching of CPR has been centered on face-to-face simulation with manikins, combining direct instruction, guided practice, and observation-based assessment.^[4]^ Nevertheless, the incorporation of digital technologies has allowed exploring new technologies that favor accessibility, the standardization of content, and the automated measurement of performance.^[5]^ In particular, immersive training, supported in interactive platforms, allow repeating procedures, receiving immediate feedback, and assessing knowledge in a structured manner.^[6]^

Despite these advances, significant challenges persist in the training of complex clinical scenarios, such as the care of patients with hypothermia and an aerial evacuation with a helicopter. These contexts present logistic and safety limitations that make training in the classroom with traditional simulation difficult. Accidental hypothermia, especially in its moderate and severe forms, alters the physiological response to resuscitation, reduces the efficacy of standard maneuvers, and requires specific clinical decisions, such as the differential use of electrical discharges or transport to centers with extracorporeal membrane oxygenation capacity.^[7]^ The ERC guides have specific recommendations for cardiorespiratory arrest in hypothermia contexts, underlining the importance of adapted and realistic training.^[1,3,7]^

VR-based simulation has emerged as a promising strategy for realistically representing these extreme settings, allowing the user to become immersed in highly-complex situations that would be difficult or impossible to replicate in person.^[7]^ In addition, these platforms allow assessing the technical performance, as well as the making of clinical decisions, in real time, which broadens its training potential.^[8]^

The aim of the present study was to analyze the performance and experience of health professionals who participated in advanced life support (ALS) training using an immersive clinical simulation exclusively based on VR, centered on the making of decisions when faced with a hypothermia scenario in a ski resort and during the helicopter transfer. The hypothesis is that a training intervention exclusively based on immersive virtual reality (VR) will allow for the identification of knowledge gaps with respect to hypothermia in extreme settings, and will be positively rated by the participants in terms of usability, perceived realism, global satisfaction, and confidence in the making of clinical decisions.

## 2. Materials and methods

### 2.1. Study design

Cross-sectional observational study with an analytical component, designed to evaluate the simulated clinical performance and the subjective perception of health professionals after training in basic life support using immersive clinical simulation with VR goggles. The description of the study follows the methodological recommendations of the STROBE statement for observational studies, and the SAMPL guide for statistical presentation in biomedical sciences.^[9,10]^

### 2.2. Context and study setting

The intervention was performed within the framework of the Refresher Course in Ultrasound and Emergencies of the Spanish Society of Family and Community Medicine (semFYC), held in Las Palmas de Gran Canaria on February 6, 7, and 8, 2025. The activity took place in a setting that was specially designed for clinical simulation, adapted with individual stations equipped with VR technology. The study was approved by the Research Ethics Committee of the University UCAM (Universidad Católica de Murcia, Spain), with code CE112309.

### 2.3. Participants

All the course participants who worked as health professionals were invited to participate. Only those who completed the entire simulation experience, as well as the assessment questionnaires, were included. The inclusion criteria were: being active in the area of health, and signing the informed consent. Participants with technical limitations or photosensitivity conditions that could make the use of VR technology difficult were excluded. The sampling was non-probabilistic, by convenience.

### 2.4. Design of the scenario and technology utilized

**Figure 1.**
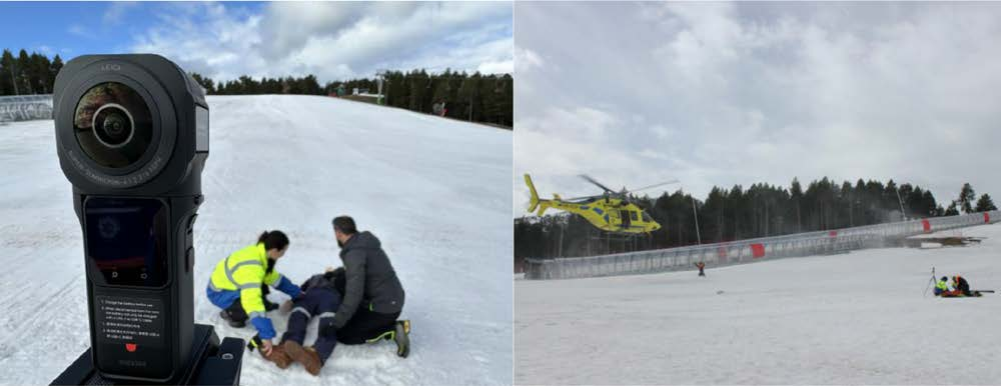
Screenshots from the filming of the immersive clinical set on the Pals ski slope (Andorra).

The clinical scenario was developed by a multidisciplinary team from the Prehospital Emergency Research Network (RINVEMER), in collaboration with the Andorran Health Service and members of the ERC. The recording took place at the Pals ski resort, in Andorra (Fig. 1), using 4 360° Insta360 X4^®^ cameras (8K resolution, FlowState stabilization, built-in noise-canceling microphone and 2 135° wide-angle lenses). The complete simulation video, in flat format (2D) is publicly available in the following link: https://youtu.be/R5SVzCW-8uI

The editing of the content was performed with the Wonda VR Studio platform, which allowed integrating interactive elements, structuring the clinical narrative, and synchronizing the decision with immediate feedback.

The experience was reproduced with the Meta Quest 3^®^ VR goggles, with an OLED display (2064 × 2208 per eye), 120 Hz refresh rate, 110° field of view, integrated eye and hand tracking, and advanced haptic controllers. This technology allowed for an independent immersion without the need for cables or connection to external computers.

### 2.5. Intervention procedure

Each participant individually accessed the scenario, in which a sequence of 16 clinical decisions were presented related with the initial care of a cardiorespiratory arrest (Supplementary Material 1, Supplemental Digital Content, https://links.lww.com/MD/R119). The platform automatically recorded the answers and provided immediate feedback at the end. No directed debriefing was performed to avoid influencing the spontaneous perception of the users.

### 2.6. Variables and measurement instruments

The variables collected were:

- **Sociodemographic and professional**: Age, sex, profession, work experience, work setting, previous CPR training, and familiarity with VR technologies.
- **Performance in the simulation**: percentage of correct answers in the clinical scenario.
- **Perception of the experience**: assessed through Likert scales (1–5) for perceived usefulness, satisfaction and confidence; and analogous scale (1–10) for realism.
- **Usefulness of the system**: measured through the standardized SUS (System Usability Scale) questionnaire, scored from 0 to 100.

### 2.7. Control of biases

The participation was voluntary and anonymous. To minimize the biases of social desirability and observation, the recording of the answers was automatically performed by the system, without the direct intervention of the research team. No economic incentives nor additional training before using the system were offered. As a stimulus, a symbolic gift was raffled off (commemorative cups) between the participants who completed the experience, without this conditioning the analysis nor the study results.

### 2.8. Sample size

The sample was composed by 101 participants. This size allowed estimating proportions with an error of ± 10% at a 95% confidence level and to detect correlations of at least *R* = 0.3 with a statistical power of 80%.

### 2.9. Data management and statistical analysis

The quantitative variables were summarized through means and standard deviations (SD) when they showed a normal distribution, or mean and interquartile range (IQR) when not. The normality of the variables was evaluated through the Shapiro–Wilk test.

The correlations between the variables were analyzed through Pearson or Spearman coefficients, accordingly. To compare between subgroups, the Student’s t or Mann–Whitney’s *U* tests were applied, depending on the distribution of the data. The differences with *P* <.05 were considered statistically significant. The statistical analysis was performed through the use of the SPSS V26 software (SPSS Inc., Chicago).

## 3. Results

### 3.1. Demographic characteristics of the professionals

The study counted with the participation of 101 health professionals who completed the simulation (Table 1). The mean age of the participants was 33.2 ± 9.0 years old (median 30.0, IQR 28.0–35.0), with a predominance of women (76.2%). Most of the participants were doctors (89.0%), with a mean professional experience of 7.4 ± 8.5 years (median 4.0 IQR 2.0–9.0).

**Table 1:** Detailed demographic characteristics and prior experience of participants.

Characteristic	n (%)/mean ± SD
Age (year)	33.2 ± 9.0
Sex	
Female	77 (76.2)
Male	24 (23.8)
Profession	
Doctor	89 (89.0)
Other health professional	11 (11.0)
Professional experience (years)	7.4 ± 8.5
Workplace	
Health center (primary care)	63 (65.6)
Hospital (Emergency or ICU)	16 (16.7)
Outpatient emergency services	5 (5.2)
Other	12 (12.5)
Experience in emergencies (years)	3.9 ± 4.8
CPR training (last 2 yr)	
1 course	34 (34.0)
2–3 courses	32 (32.0)
>3 courses	19 (19.0)
None	15 (15.0)
Frequency of CPR in the clinical practice (in the last year)	
Nonee	47 (47.0)
1–2 times	38 (38.0)
3–5 times	12 (12.0)
>5 times	3 (3.0)
Experience with virtual reality	
None previously	63 (63.0)
I have tried VR for entertainment	30 (30.0)
I have tried VR for health care training	6 (6.0)
I have used VR in advanced medical training	1 (1.0)

CPR = cardiopulmonary resuscitation, ICU = intensive care unit, SD = standard deviation, VR = virtual reality.

With respect to the work setting, 65.6% worked at primary care centers, while 16.7% worked in hospitals (mainly emergency services). The specific experience in emergency services was 3.9 ± 4.8 years (median 3.0, IQR 1.0–4.0).

As for the previous training on cardiopulmonary resuscitation, 85% of the participants had taken at least 1 course in the last 2 years, although almost half (47.0%) did not have to perform CPR maneuvers in their clinical practice in the last year. It is notable that most of the participants (63%) did not have previous experience with VR technologies, while 30.0% had only used it for recreational purposes.

### 3.2. Results from the VR experience

In the global analysis of the clinical scenario with VR, the mean percentage of correct decisions was 69.4% (SD = 15.2), with a median of 73% and a range between 45% and 94%. These results showed a generally moderate performance, with an intermediate variability between the different clinical decisions proposed.

**Figure 2.**
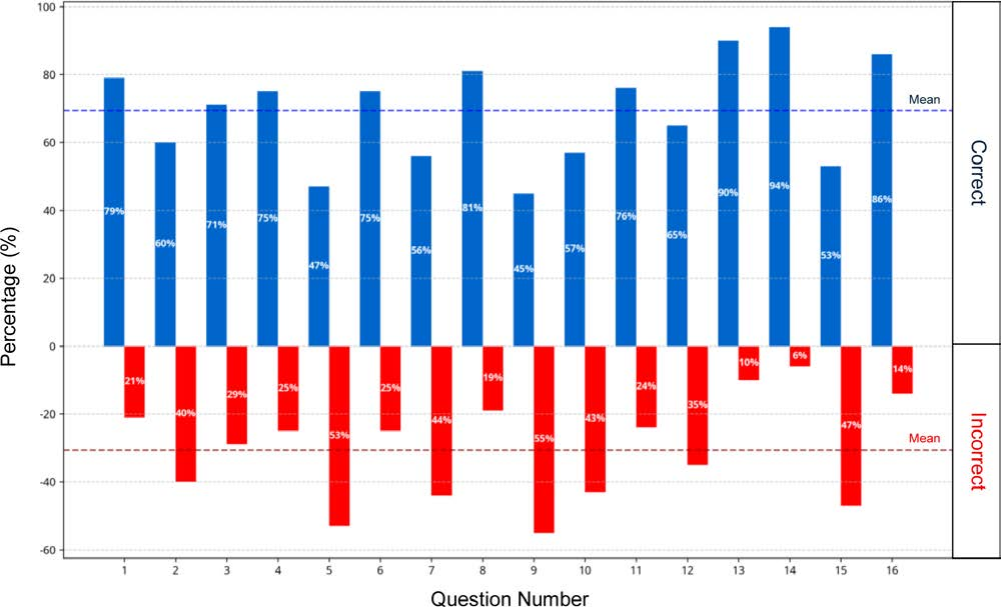
Percentage of correct and incorrect answers per question in the hypothermia scenario.

Figure 2 shows the percentages of correct and incorrect answers for each of the 16 questions of the scenario. The question with the highest percentage of correct answers was number 14 (“*What technique should be applied when approaching the patient and how should it affect the time to hospital?*”) with an accuracy of 94%, while the lowest percentage was number 9 (“*What action should be taken if ventricular fibrillation continues after 3 defibrillations?”*), with an accuracy of 45%. This distribution suggests that although the scenario showed a moderate level of difficulty in general, there were specific elements that were particularly challenging for the participants.

### 3.3. Perception of the usefulness of VR and its impact on knowledge and confidence

The participants scored VR as a training tool very positively. Table 2 shows mean scores higher than 4.7/5 in all the perceptions of usefulness, with the ability to reproduce difficult clinical scenarios (M = 4.82, SD = 0.44) and increased motivation (M = 4.80, SD = 0.45) as the most highly valued aspects. The realism of the simulation received 8.87/10 points (SD = 1.26).

**Table 2:** Perception of usefulness of VR.

Variable	Mean ± SD	95% CI	Median (IQR)
VR is useful in medical training	4.72 ± 0.61	(4.60–4.84)	5.0 (5.0–5.0)
VR improves understanding of life support	4.71 ± 0.59	(4.59–4.82)	5.0 (5.0–5.0)
Immersive technology increases motivation	4.80 ± 0.45	(4.71–4.89)	5.0 (5.0–5.0)
VR reproduces difficult clinical scenarios	4.82 ± 0.44	(4.73–4.90)	5.0 (5.0–5.0)
Realistic and immersive VR experience	4.74 ± 0.56	(4.63–4.85)	5.0 (5.0–5.0)
Simulation realism	8.87 ± 1.26	(8.62–9.12)	9.0 (8.0–10.0)
VR improves safety and confidence	4.47 ± 0.78	(4.32–4.62)	5.0 (4.0–5.0)
Improvement in knowledge and skills	4.17 ± 0.92	(3.99–4.36)	4.0 (4.0–5.0)
Overall satisfaction with the experience	4.65 ± 0.58	(4.54–4.77)	5.0 (4.0–5.0)

CI = confidence interval, IQR = interquartile range, SD = standard deviation, VR = virtual reality.

**Figure 3.**
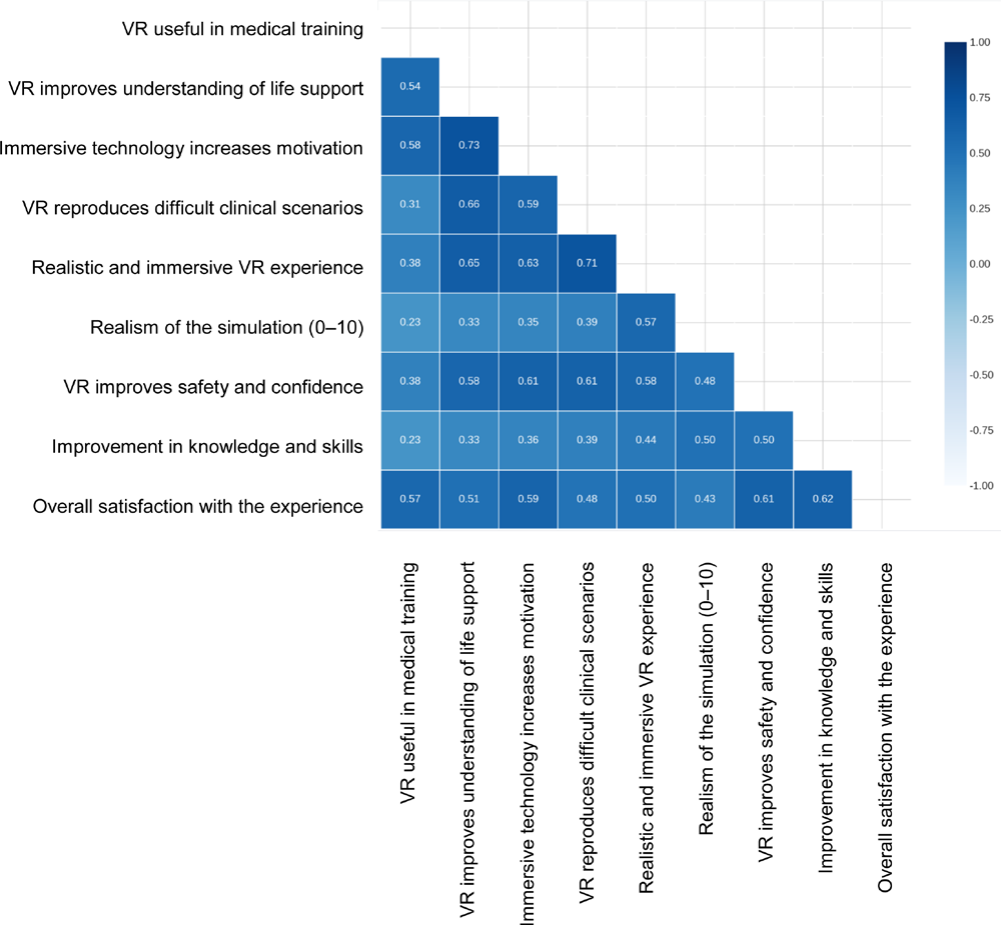
Correlation matrix between variables of perceived usefulness of VR and impact on clinical knowledge and confidence. VR = virtual reality.

As for the impact of the training, the participants reported an improvement in their confidence in the making of decisions (M = 4.47, SD = 0.78) and their CPR knowledge and skills (M = 4.17, SD = 0.92). Figure 3 shows significant correlations between key variables. The strongest associations were observed between immersive technology and the understanding of concepts (*R* = 0.73, *P* <.001), and between realistic experience and reproduction of clinical scenarios (*R* = 0.71, *P* <.001). The correlations between general satisfaction and improvement in knowledge (*R* = 0.62, *P* <.001), and between realism and improvement in knowledge (*R* = 0.50, *P* <.001) were also notable.

No significant differences were observed according to the previous experience with VR, suggesting that the tool was equally valued by all the participants, independently of their familiarity with this technology.

### 3.4. Usefulness of the virtual reality system

**Figure 4.**
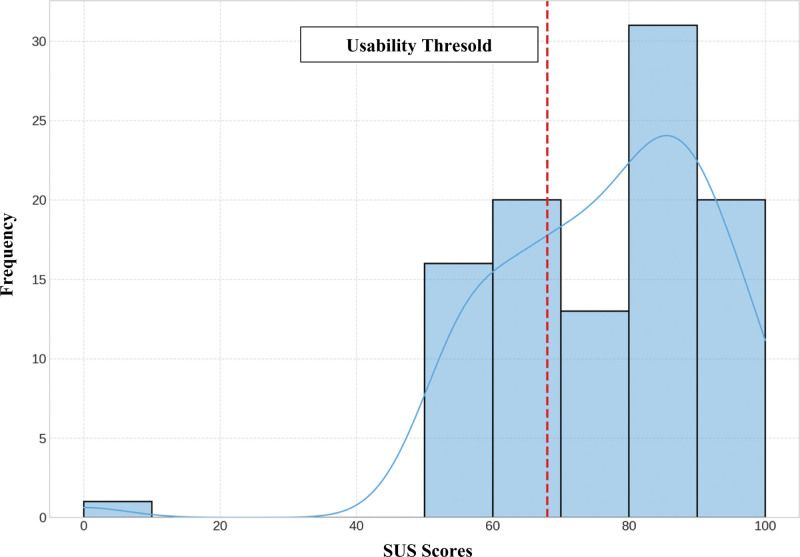
Distribution of SUS scores for the evaluated VR system. SUS = system usability scale, VR = virtual reality.

The assessment of usability of the VR system through the SUS (*System Usability Scale*) questionnaire provided a mean score of 76.68 (SD = 16.06), which indicates a “Good” level of usability according to the standard interpretation criteria (Fig. 4). This score exceeded the acceptability threshold of 68 points. A total of 63.3% of the participants scored the system as “Good” (25.7%) or “Excellent” (37.6%), while only 3.0% considered it “Unacceptable.” The median was 80.56 (IQR 66.67–88.89). The aspects that obtained the best scores were comfortableness of use (M = 3.55/4) and the intent of frequent use (M = 3.48/4). The aspect with the lowest score was the need for technical support (M = 2.01/4).

## 4. Discussion

### 4.1. Main results and interpretation

The present study provides preliminary evidence on the applicability and acceptance of immersive VR-based education for training in clinical decisions related to BLS, in a hypothermia context during helicopter transfer. The global performance observed (69.4%), and the positive perception expressed by the participants, show an intervention with an educational potential in complex clinical scenarios.

The ERC guides for special situations recognize the need for specific hypothermia protocols, underlining that the making of decisions in these contexts requires the precise understanding of physiological and therapeutic modifications involved.^[11]^ The simulation of these types of scenarios through traditional methods tends to be complex, costly, and difficult to replicate. In this sense, VR represents a promising alternative to train health professionals in extreme settings, with a low risk and high contextual fidelity.^[12,13]^

Likewise, the ERC education guides stress that innovative methods must be evaluated not only in knowledge acquisition terms, but also due to their ability to have an impact on the retention, the transfer to the real-world clinical setting, and the self-efficacy of the professionals.^[14]^ In our study, the participants showed high levels of satisfaction, perceived realism, and confidence in the knowledge acquired, with these aspects also pointed out as key in the acceptance of technology according to contemporary pedagogic models.^[15,16]^

The recent literature highlights that immersion, emotional commitment, and the sense of presence in virtual settings act as facilitators of meaningful learning, especially in complex or critical tasks such as cardiopulmonary resuscitation.^[17–19]^ In addition, when a coherent clinical narrative is utilized, such as the one posed in the present study, situated learning is promoted, and the making of decisions in real time is optimized.^[20]^

From a technical perspective, the use of independent devices, such as VR goggles, was positively rated in previous studies due to their ease of use, portability, and low implementation cost in learning environments without the need for a specialized infrastructure.^[21,22]^ These findings reinforce the logistic viability of the intervention performed.

### 4.2. Comparison with the literature

Fijačko et al documented the changes in the positioning of ILCOR with respect to the use of immersive technologies in CPR training: from a weak recommendation based on low-quality evidence, towards a greater openness to controlled effectiveness studies.^[23]^

On their part, Greif et al summarized how the current educational recommendations advocate for a structured use of VR and AR technologies, as long as they integrate with clear learning objectives and efficient feedback mechanisms.^[14]^

The positive perception of the participants in our experience coincides with the findings from multiple recent studies that show that VR not only increases motivation, but also improves the self-perception of competence, and the will to face real clinical scenarios.^[24,25]^ In the area of health training, these variables have shown to be predictors of both clinical performance and adherence to guides in real-world contexts.^[26,27]^

### 4.3. Strengths and limitations

Among the main strengths of the study, the design of the scenario about a situation, based on a real clinical situation, recorded with 360° technology in an operational setting, is notable. Likewise, the use of objective performance metrics, complemented with standardized perception tools (such as the SUS questionnaire), was highly valued. The collection of data was automatic and anonymous, which minimizes observer and social desirability biases.

As limitations, we must mention the convenience sampling, the absence of a group control, and the lack of longitudinal follow-up that would allow us to assess the retention of knowledge or its transfer to a health care setting. In addition, the performance analysis was exclusively based on the making of clinical decisions, without considering psychomotor skills.

### 4.4. Implications and future projections

The evidence collected supports the progressive inclusion of VR as a complementary tool in continuing training programs on emergencies, especially in low-frequency and high-complexity clinical situations. This integration must be performed with solid pedagogic criteria, aligned with international recommendations, and by assessing its impact on learning, the safety of the patient, and the institutional sustainability.^[14]^

Future studies must focus on controlled designs that compare VR with traditional methods, the assessment of retention skills, its clinical repercussion, and its practical applicability. In addition, it is necessary to explore scalable implementation models, especially in health systems with limited resources.

## 5. Conclusions

The study shows that a training intervention based on immersive VR, applied to a complex clinical scenario such as hypothermia in the context of an aerial (helicopter) transfer, is feasible, well-accepted by the health professionals, and useful for training on decision making in ALS.

The results obtained support the pedagogic and technical viability of incorporating this modality in continuing emergency training programs. The positive perception of the participants, along with the global performance reached, suggests that VR can be an efficient tool for favoring situated learning, especially in low-frequency and high-complexity clinical situations, where traditional simulation faces important limitations.

Nevertheless, additional controlled studies are needed that assess its medium and long-term impact, as well as its repercussion on clinical practice and the safety of the patient.

## Acknowledgments

The authors would like to thank the professionals at the VR Lab at the UCAM: A. Lanchares, A. Pedreño, and J. Alonso. We would also like to thank the “Servei Urgent Mèdic” of the “Servei Andorrà d’Atenció Sanitaria” and “Heliand” for their collaboration and assistance in creating the scenario used.

## Author contributions

**Conceptualization:** Marina Gómez Sánchez, Carmen Amalia López López, Robert Greif, Federico Semeraro, Manuel Pardo Rios, Daniel Guillén Martínez.

**Data curation:** Marina Gómez Sánchez, Carmen Amalia López López, Manuel Pons Claramonte, Daniel Guillén Martínez.

**Formal analysis:** Marina Gómez Sánchez, Carmen Amalia López López, Ana Belén Ocampo Cervantes, Manuel Pons Claramonte.

**Funding acquisition:** Ana Belén Ocampo Cervantes, Manuel Pardo Rios, Daniel Guillén Martínez.

**Investigation:** Robert Greif, Federico Semeraro, Ana Belén Ocampo Cervantes, Manuel Pardo Rios.

**Methodology:** Federico Semeraro, Petronila Mireia Alcazar Artero.

**Project administration:** Manuel Pardo Rios.

**Resources:** Ana Belén Ocampo Cervantes.

**Supervision:** Marina Gómez Sánchez, Carmen Amalia López López, Ana Belén Ocampo Cervantes, Sergio Nieto Caballero, Petronila Mireia Alcazar Artero.

**Validation:** Marina Gómez Sánchez, Carmen Amalia López López, Sergio Nieto Caballero.

**Visualization:** Petronila Mireia Alcazar Artero.

**Writing – original draft:** Marina Gómez Sánchez, Carmen Amalia López López, Robert Greif, Ana Belén Ocampo Cervantes, Sergio Nieto Caballero, Daniel Guillén Martínez.

**Writing – review & editing:** Robert Greif, Federico Semeraro, Sergio Nieto Caballero, Petronila Mireia Alcazar Artero, Manuel Pardo Rios, Daniel Guillén Martínez.

## Supplementary Material


