# Supplementary material for: Evaluation of advanced life support training through virtual reality: A cross-sectional observational study in a hypothermia and helicopter evacuation scenario
Source: Medicine (Baltimore). 2026 Jan 16;105(3):e47135. doi: 10.1097/MD.0000000000047135 (PMC12826293; doi:10.1097/MD.0000000000047135)
Supplement: Supplementary file 1 [file medi-105-e47135-s001.docx]

**Supplementary Material 1.** Questionnaire used in CPR and hypothermia training using virtual reality.

1. **What should the rescuer do upon arrival?**
   · a) Protect, alert, and rescue
   · b) Call 112
   · c) Request a medical helicopter
   · d) Shake the patient to see if they are conscious
2. **In the event of a person losing consciousness in a mountainous environment, what should the rescuer do?**
   · a) Open the airway
   · b) Call 112
   · c) Start chest compressions
   · d) Place the victim in the lateral recovery position
3. **After verifying that the patient is unconscious and not breathing, what should the rescuer do??**
   · a) Start chest compressions
   · b) Give 5 rescue breaths
   · c) Give 2 rescue breaths as for drowning victims
   · d) Wait for medical services to arrive
4. **What is the appropriate rate for chest compressions and ventilations during CPR in hypothermic patients?**
   · a) 15:2, as in pediatric resuscitation
   · b) 30:2, same as in normothermia
   · c) 10:1, since ventilation is more important
   · d) 40:2, to improve blood oxygen levels
5. **According to the protocol, how should we proceed when treating a patient who shows signs of hypothermia?**
   · a) Remove all of the patient's clothing to assess his or her condition.
   · b) Remove only the clothing necessary to assess the patient's condition.
   · c) Keep the patient's clothing to a minimum level of exposure.
   · d) Wait for hospital staff to arrive to perform the assessment.
6. **Ventricular fibrillation is detected on the monitor, what is the most appropriate action??**
   · a) Continue chest compressions.
   · b) Check pulse
   · c) Apply defibrillation
   · d) Apply cardioversion
7. **If a patient with a temperature <30°C is in cardiac arrest, what specific recommendation does the algorithm mention regarding defibrillation?**
   · a) Do not perform defibrillation until the temperature is greater than 32°C
   · b) Perform defibrillation immediately without waiting for rewarming
   · c) After applying a first shock, we wait to rewarm the patient before trying again.
   · d) Defibrillation is not necessary in patients with severe hypothermia.
8. **What should be done in case of non-reactive mydriasis or signs such as lividity or rigor mortis in a hypothermic patient?**
   · a) Start the death protocol, as these are signs of certain death.
   · b) Continue CPR and treatment until the patient is rewarmed.
   · c) Stop CPR, as non-reactive mydriasis is irreversible.
   · d) Perform ventilations only and wait for improvement
9. **What action should be taken if ventricular fibrillation continues after three defibrillations?**
   · a) Continue with more shocks without waiting
   · b) Delay further attempts until body temperature is >30°C
   · c) Administer adrenaline immediately
   · d) Start immediate reheating
10. **After evaluating the patient's core temperature, if the temperature is less than 32ºC, what procedure is most recommended?**
    · a) Active external reheating (thermal blankets, room heating)
    · b) Internal rewarming with warm intravenous fluids
    · c) Administration of warm oxygen
    · d) Begin advanced resuscitation maneuvers
11. **Under what conditions should adrenaline not be administered to a hypothermic patient?**
    · a) Body temperature <30°C
    · b) Body temperature >30°C
    · c) During the transfer
    · d) Only if there is ventricular fibrillation
12. **How should adrenaline administration be adjusted when body temperature is >30°C?**
    · a) Increase administration intervals to every 6–10 minutes
    · b) Decrease administration intervals to every 2–3 minutes
    · c) Administer adrenaline continuously
    · d) Do not administer adrenaline
13. **What is the key intervention for hypothermic patients before transfer to a hospital?**
    · a) Administer adrenaline immediately
    · b) CPR, immobilization and thermal isolation
    · c) Start immediate reheating
    · d) Continuous CPR
14. **What technique should be applied when managing the patient and how should it affect the time to hospital?**
    · a) Apply WRAP and significantly delay arrival at the hospital.
    · b) Apply WRAP without causing a significant delay in arrival at the hospital.
    · c) Apply WRAP only if rapid transport cannot be performed.
    · d) Do not apply WRAP to avoid delays.
15. **Which device is recommended for prolonged transport or difficult terrain?**
    · a) Manual defibrillator
    · b) Cardiocompressor
    · c) Manual ventilator
    · d) Temperature stabilizer
16. **During transfer, if possible, what type of CPR should be performed on patients in cardiac arrest due to hypothermia?**
    · a) Intermittent CPR
    · b) Continuous CPR
    · c) Breaths only
    · d) Chest compressions only
